# TRPV4 is a Prognostic Biomarker that Correlates with the Immunosuppressive Microenvironment and Chemoresistance of Anti-Cancer Drugs

**DOI:** 10.3389/fmolb.2021.690500

**Published:** 2021-06-28

**Authors:** Kai Wang, Xingjun Feng, Lingzhi Zheng, Zeying Chai, Junhui Yu, Xinxin You, Xiaodan Li, Xiaodong Cheng

**Affiliations:** ^1^ Department of Gynecologic Oncology, Women’s Hospital, School of Medicine, Zhejiang University, Hangzhou, China; ^2^ Department of Obstetrics and Gynecology, Taizhou Hospital of Zhejiang Province Affiliated to Wenzhou Medical University, Linhai, China

**Keywords:** TRPV4, tumor immune microenvironment, pan-cancer, tumor immunosuppressive status, tumor-associated macrophage

## Abstract

**Background:** Transient receptor potential cation channel subfamily V member 4 (TRPV4) has been reported to regulate tumor progression in many tumor types. However, its association with the tumor immune microenvironment remains unclear.

**Methods:** TRPV4 expression was assessed using data from The Cancer Genome Atlas (TCGA) and the Genotype-Tissue Expression (GTEx) database. The clinical features and prognostic roles of TRPV4 were assessed using TCGA cohort. Gene set enrichment analysis (GSEA) of TRPV4 was conducted using the R package clusterProfiler. We analyzed the association between TRPV4 and immune cell infiltration scores of TCGA samples downloaded from published articles and the TIMER2 database. The IC50 values of 192 anti-cancer drugs were downloaded from the Genomics of Drug Sensitivity in Cancer (GDSC) database and the correlation analysis was performed.

**Results:** TRPV4 was highly expressed and associated with worse overall survival (OS), disease-specific survival (DSS), disease-free interval (DFI), and progression-free interval (PFI) in colon adenocarcinoma (COAD) and ovarian cancer. Furthermore, TRPV4 expression was closely associated with immune regulation-related pathways. Moreover, tumor-associated macrophage (TAM) infiltration levels were positively correlated with TRPV4 expression in TCGA pan-cancer samples. Immunosuppressive genes such as PD-L1, PD-1, CTLA4, LAG3, TIGIT, TGFB1, and TGFBR1 were positively correlated with TRPV4 expression in most tumors. In addition, patients with high expression of TRPV4 might be resistant to the treatment of Cisplatin and Oxaliplatin.

**Conclusion:** Our results suggest that TRPV4 is an oncogene and a prognostic marker in COAD and ovarian cancer. High TRPV4 expression is associated with tumor immunosuppressive status and may contribute to TAM infiltration based on TCGA data from pan-cancer samples. Patients with high expression of TRPV4 might be resistant to the treatment of Cisplatin and Oxaliplatin.

## Background

TRPV4 is a broadly expressed polymodally activated ion channel. It is reported to be involved in tumor progression in several tumor types, playing different or even opposite roles therein. For example, overexpression of TRPV4 promotes epithelial to mesenchymal transition in breast cancer cells (Huang et al., 2021). TRPV4 also promotes metastasis of endometrial cancer cells by regulating the RhoA/ROCK1 pathway (Li et al., 2020). In contrast, TRPV4 activation inhibits glioma progression (Huang et al., 2021). Despite these reports, the role of TRPV4 in most tumor types remains unclear.

A number of studies indicate that the tumor immune microenvironment (TIME) has clinicopathological significance in predicting therapeutic effect and prognosis in tumor patients (Hubert et al., 2021; Li et al., 2021; Liu et al., 2021). It has been confirmed that solid tumors are composed of malignant, non-malignant, hematopoietic, and mesenchymal cells. Among the non-malignant cells, tumor-associated macrophages (TAMs) play an essential role in promoting tumor progression (Deng et al., 2021; Gill et al., 2021). High TAM levels in tumor tissues influence the immune escape status of tumors, rendering immunotherapy ineffective (Bi et al., 2021). Thus, exploring the relationship between TRPV4 and TAM is helpful to understand the role of TRPV4 in tumor progression.

In our study, we assessed TRPV4 expression using data from The Cancer Genome Atlas (TCGA) and the Genotype-Tissue Expression (GTEx) database and found that TRPV4 was differentially expressed in 25 tumor types. Furthermore, in patients with colon adenocarcinoma (COAD) or ovarian cancer, high TRPV4 expression was associated with worse overall survival (OS), disease-specific survival (DSS), disease-free interval (DFI), and progression-free interval (PFI). TRPV4 is predicted to influence immune regulation-related pathways. We further assessed the association between TRPV4 expression and immune cell infiltration scores and immunosuppressive genes in TCGA pan-cancer samples and found that TRPV4 expression was positively correlated with TAM infiltration and immunosuppressive genes such as PD-L1, PD-1, CTLA4, LAG3, TIGIT, TGFB1, and TGFBR1 in pan-cancer samples. In addition, we further assessed the influence of TRPV4 on resistance of patients to anti-cancer drugs.

Our study explored the role of TRPV4 in TCGA pan-cancer and further highlight a potential function whereby TRPV4 may regulate TAM infiltration and tumor immunosuppressive microenvironment.

## Methods

### Data Sources

TCGA and GTEx expression and clinical data were obtained from the UCSC Xena database (https://xenabrowser.net/datapages/). DNA copy number and methylation data were downloaded from the cBioPortal database (https://www.cbioportal.org/). The IC_50_ values of drugs and gene expression profiles in the relative cell lines were downloaded from the Genomics of Drug Sensitivity in Cancer (GDSC) database (https://www.cancerrxgene.org/). The number of samples used in this study as follows:

1. TCGA tumor samples: ACC-77; BLCA-407; BRCA-1098; CESC-306; CHOL-36; COAD-288; DLBC-47; ESCA-182; GBM-163; HNSC-520; KICH-66; KIRC-531; KIRP-289; LAML-173; LGG-522; LIHC-371; LUAD-515; LUSC-498; MESO-87; OV-427; PAAD-179; PCPG-182; PRAD-496; READ-92; SARC-262; SKCM-469; STAD-414; TGCT-137; THCA-512; UCEC-181; THYM-119; UCS-57; UVM-79.
2. TCGA normal samples: ACC-0; BLCA-19; BRCA-113; CESC-3; CHOL-9; COAD-41; DLBC-0; ESCA-13; GBM-0; HNSC-44; KICH-25; KIRC-72; KIRP-32; LAML-0; LGG-0; LIHC-50; LUAD-59; LUSC-50; MESO-0; OV-0; PAAD-4; PCPG-3; PRAD-52; READ-10; SARC-2; SKCM-1; STAD-36; TGCT-0; THCA-59; UCEC-13; THYM-0; UCS-0; UVM-0.
3. GTEx normal samples: ACC-128; BLCA-9; BRCA-179; CESC-10; CHOL-0; COAD-308; DLBC-444; ESCA-653; GBM-1152; HNSC-0; KICH-28; KIRC-28; KIRP-28; LAML-70; LGG-1152; LIHC-110; LUAD-288; LUSC-288; MESO-0; OV-88; PAAD-167; PCPG-0; PRAD-167; READ-308; SARC-0; SKCM-812; STAD-174; TGCT-165; THCA-279; UCEC-78; THYM-444; UCS-78; UVM-0.

### Data Analysis Tools

Copy number alteration (CNA) and mutation status of TRPV4 in TCGA pan-cancer samples were analyzed using the cBioPortal database. The TIMER2 (http://timer.cistrome.org/) database was used to analyze the correlation between immune cell infiltration and TRPV4. R packages ggplot2, ggpubr, and gridExtra were used to draw TRPV4 expression patterns in the R software (3.6.2). R packages survival and survminer were used to perform Kaplan-Meier survival analysis and univariate Cox regression analysis, respectively. Gene set enrichment analysis (GSEA) was performed using the R package clusterProfiler.

### TIME Analysis

Immune cell infiltration correlation analysis was performed using two methods: 1) the TIMER2 database was used to analyze the correlation between immune cell infiltration and TRPV4; and 2) the immune cell infiltration scores of TCGA pan-cancer samples were downloaded from the ImmuCellAI database (http://bioinfo.life.hust.edu.cn/web/ImmuCellAI/) to perform the correlation analysis. To compare levels of immune cell infiltration, tumor samples from each tumor type were divided into two groups (high-TRPV4 and low-TRPV4 groups) according to the median expression of TRPV4 in each tumor types.

### Statistical Analysis

Data are presented as the mean ± SD. Differences between the groups were analyzed using Student’s t-test. Statistical analysis was performed using R 3.6.2. *p* < 0.05 (two-tailed) was considered statistically significant: *p < 0.05, **p < 0.01, ***p < 0.001, and ****p < 0.0001.

## Results

### TRPV4 Expression in Pan-Cancer

**FIGURE 1:**
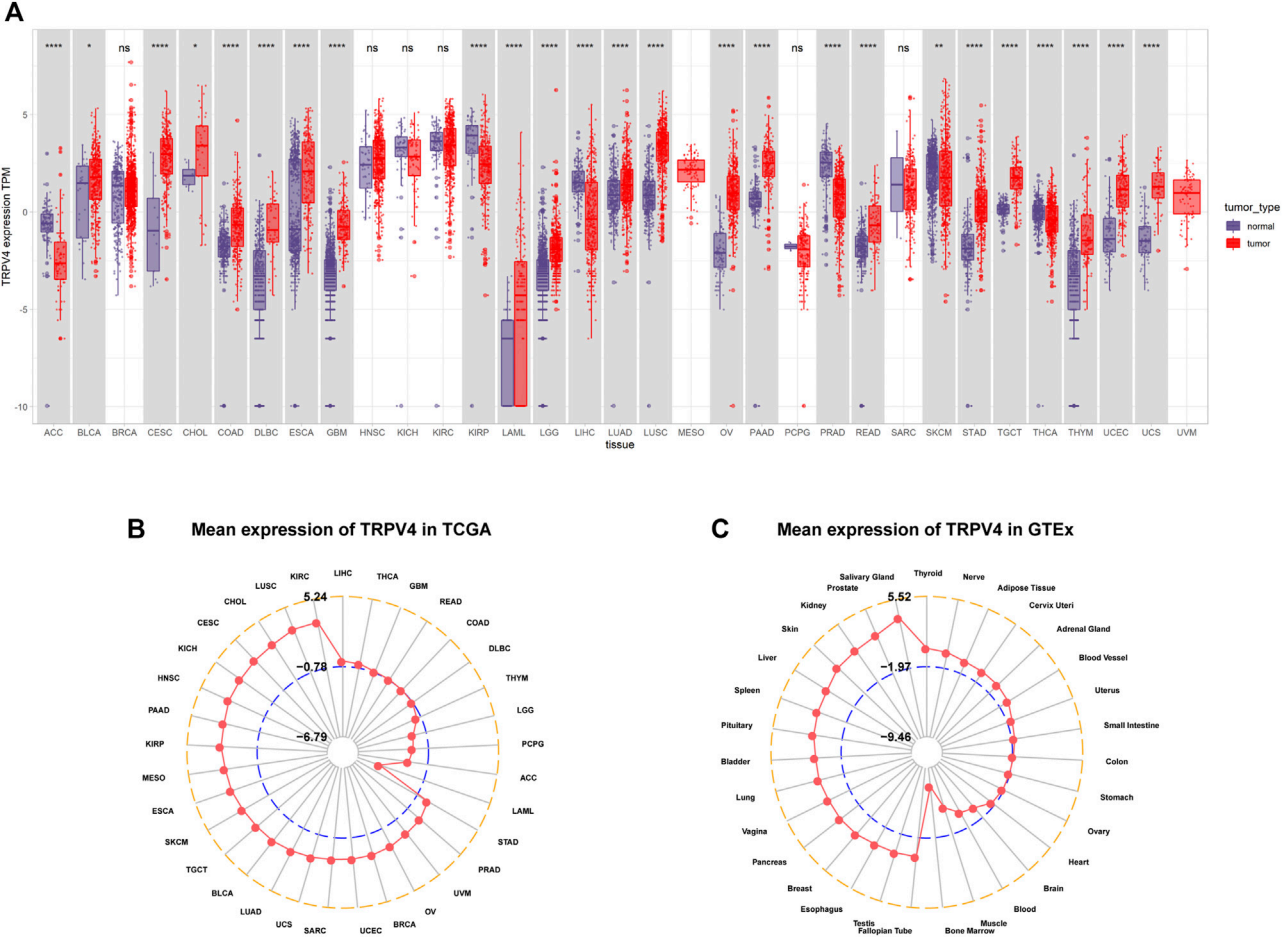
Pan-cancer TRPV4 expression analysis. **(A)** Pan-cancer TRPV4 expression between tumor tissues from TCGA and normal tissues from TCGA and the GTEx database. **(B)** Radar plot shows TRPV4 expression levels in tumor tissues from TCGA. **(C)** Radar plot shows TRPV4 expression levels in normal tissues from the GTEx database.

We first assessed TRPV4 expression in tumor tissue samples from TCGA and normal tissue samples from TCGA and the GTEx database. We found that TRPV4 was overexpressed in 19 cancer types, namely, bladder urothelial carcinoma (BLCA), cervical squamous cell carcinoma and endocervical adenocarcinoma (CESC), cholangiocarcinoma (CHOL), colon adenocarcinoma (COAD), lymphoid neoplasm diffuse large B-cell lymphoma (DLBCL), esophageal carcinoma (ESCA), glioblastoma multiforme (GBM), acute myeloid leukemia (LAML), brain low-grade glioma (LGG), lung adenocarcinoma (LUAD), lung squamous cell carcinoma (LUSC), ovarian serous cystadenocarcinoma, pancreatic adenocarcinoma (PAAD), rectum adenocarcinoma (READ), stomach adenocarcinoma (STAD), testicular germ cell tumors (TGCTs), thymoma (THYM), uterine corpus endometrial carcinoma (UCEC), and uterine carcinosarcoma (UCS). In contrast, low TRPV4 expression was observed in only six cancer types, namely, adrenocortical carcinoma (ACC), kidney renal papillary cell carcinoma (KIRP), liver hepatocellular carcinoma (LIHC), prostate adenocarcinoma (PRAD), skin cutaneous melanoma (SKCM), and thyroid carcinoma (THCA) (Figure 1A). For tumor tissue data mined from TCGA, TRPV4 expression was highest in kidney renal clear cell carcinoma (KIRC) and lowest in LAML (Figure 1B). For normal tissue data from the GTEx database, the highest TRPV4 expression was observed in the salivary gland and prostate, while the lowest expression was detected in bone marrow (Figure 1C).

**FIGURE 2:**
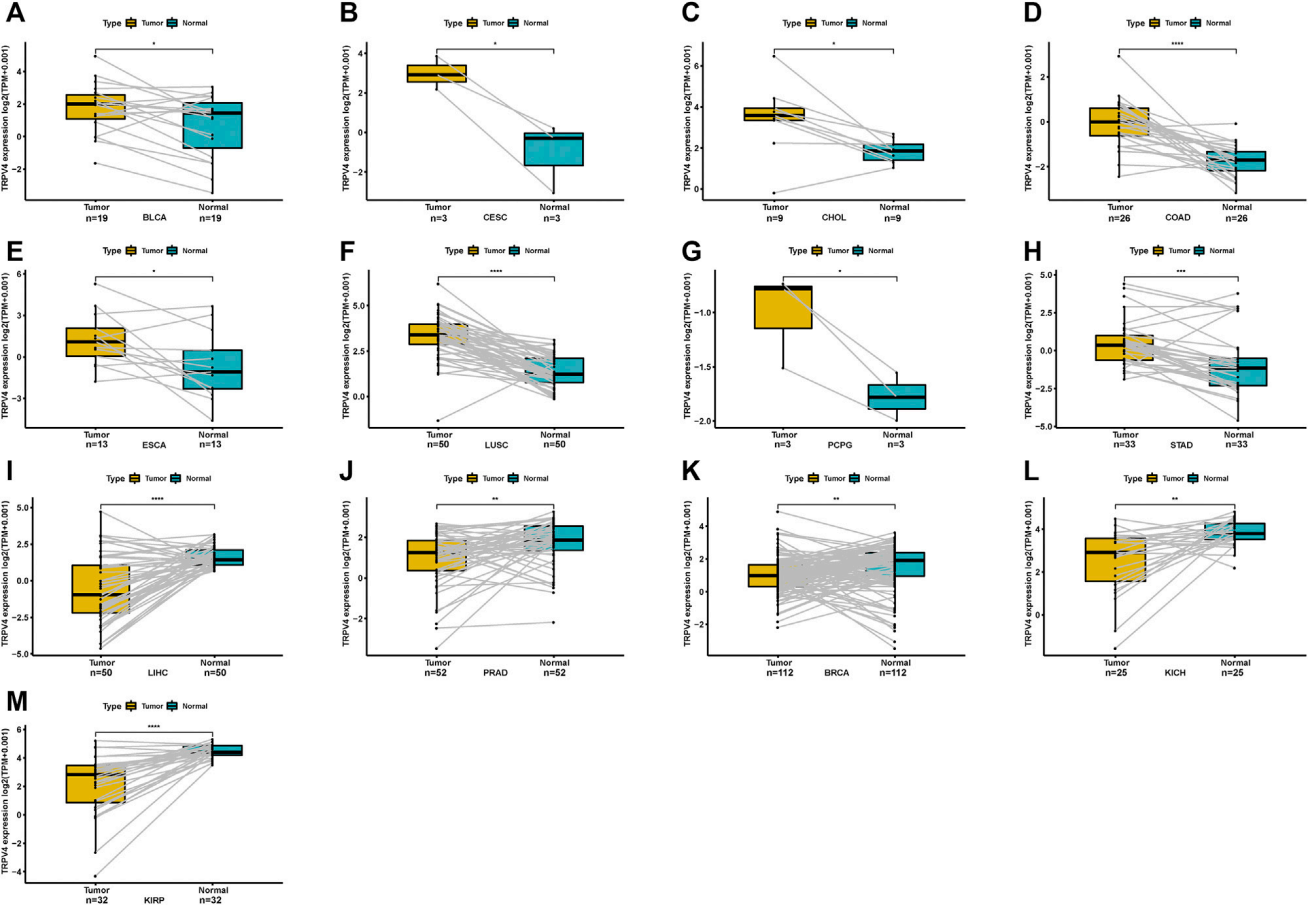
Pan-cancer TRPV4 expression in paired tumor and adjacent normal tissues. **(A–M)** Pan-cancer TRPV4 expression in paired tumor and adjacent normal tissues in indicated tumor types from TCGA.

For paired tumors and normal tissues in TCGA, TRPV4 was overexpressed in BLCA, CESC, CHOL, COAD, ESCA, LUSC, pheochromocytoma/paraganglioma (PCPG), and STAD (Figures 2A–H), while low TRPV4 expression was observed in LIHC, PRAD, breast invasive carcinoma (BRCA), kidney chromophobe (KICH), and KIRP (Figures 2I–M).

### TRPV4 Alteration Analysis

**FIGURE 3:**
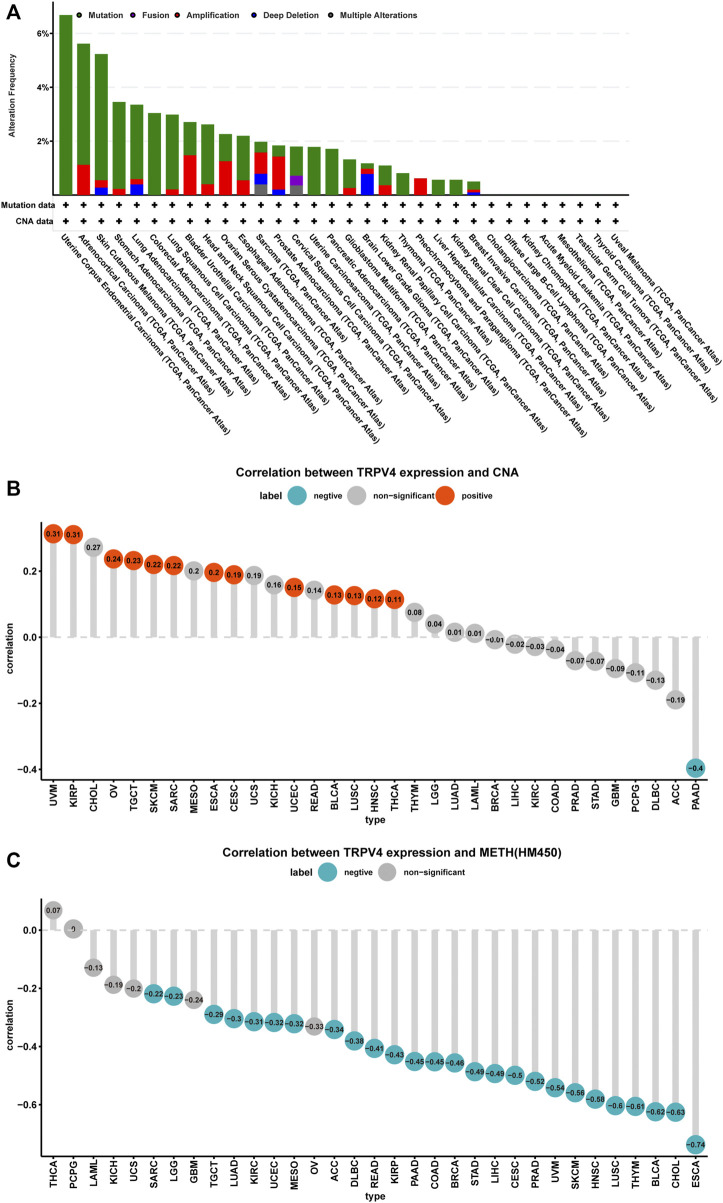
TRPV4 gene alterations in pan-cancer. **(A)** CNA and mutation status of TRPV4 in TCGA pan-cancer samples. **(B)** The correlation between TRPV4 expression and linear copy-number value in TCGA pan-cancer samples. **(C)** The correlation between TRPV4 expression and methylation levels of TRPV4 as a promoter in TCGA pan-cancer samples. The pearson’s correlation coefficient was calculated using R software.

We further explored TRPV4 gene alterations in TCGA pan-cancer samples using cBioPortal and observed that patients with UCEC and ACC presented high gene alteration frequencies, including mutations and amplifications (Figure 3A). In addition, we downloaded copy number and methylation data for TRPV4 and performed a correlation analysis. The results revealed that copy number values were positively correlated with TRPV4 expression (Figure 3B), while methylation levels were negatively correlated with TRPV4 expression in most tumor types from TCGA (Figure 3C). These results indicate that high copy number values and low methylation levels contribute to high TRPV4 expression in pan-cancer.

### Prognostic Role of TRPV4

**FIGURE 4:**
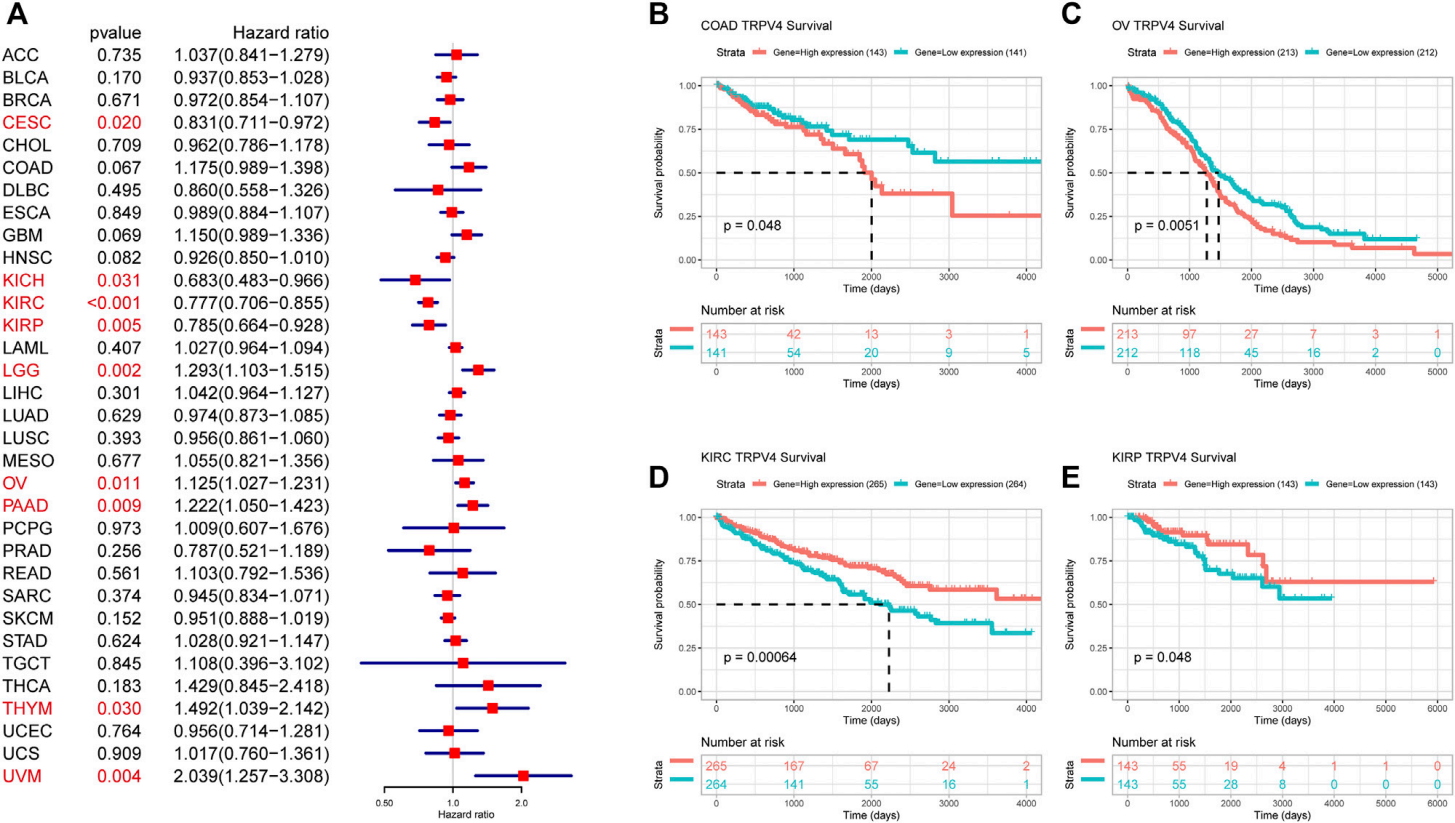
OS analysis of TRPV4 in pan-cancer. **(A)** Forest map shows the univariate Cox regression analysis results for TRPV4 in TCGA pan-cancer samples. Red indicates significant results. **(B)** Kaplan-Meier survival analysis results for TRPV4 in indicated tumor types. The median values of TRPV4 in COAD, OV, KIRC, and KIRP were respectively -0.6416, 0.9493, 3.4129, and 2.4479.

To further evaluate the significance of TRPV4 as a prognostic marker in tumor patients, we performed univariate Cox regression analysis and Kaplan-Meier survival analysis using TCGA pan-cancer data. Results from the univariate Cox regression analysis suggested that TRPV4 was a risk factor for OS in LGG, ovarian cancer, PAAD, THYM, and UVM patients, while it was a protective factor in CESC, KICH, KIRC, and KIRP (Figure 4A). Kaplan-Meier survival analysis revealed that high TRPV4 expression predicted poorer OS in patients with ACC, HNSC, KIRP, LIHC, LUAD, and UVM and better OS in patients with KIRC and KIRP (Figure 4B). We further conducted DFI, PFI, and DSS assessments using univariate Cox regression analysis. For DFI, TRPV4 was a risk factor in PAAD and a protective factor in UCEC and UCS (Supplementary Figure S1A). For PFI, TRPV4 was a risk factor in COAD, LGG, PAAD, and UVM and a protective factor in KICH, KIRC, KIRP, and UCEC (Supplementary Figure S1B). For DSS, TRPV4 was a risk factor in COAD, GBM, LGG, LUSC, ovarian cancer, PAAD, THYM, and UVM and a protective factor in CESC, KICH, KIRC, KIRP, and LUSC (Supplementary Figure S1C).

### GSEA of TRPV4

**FIGURE 5:**
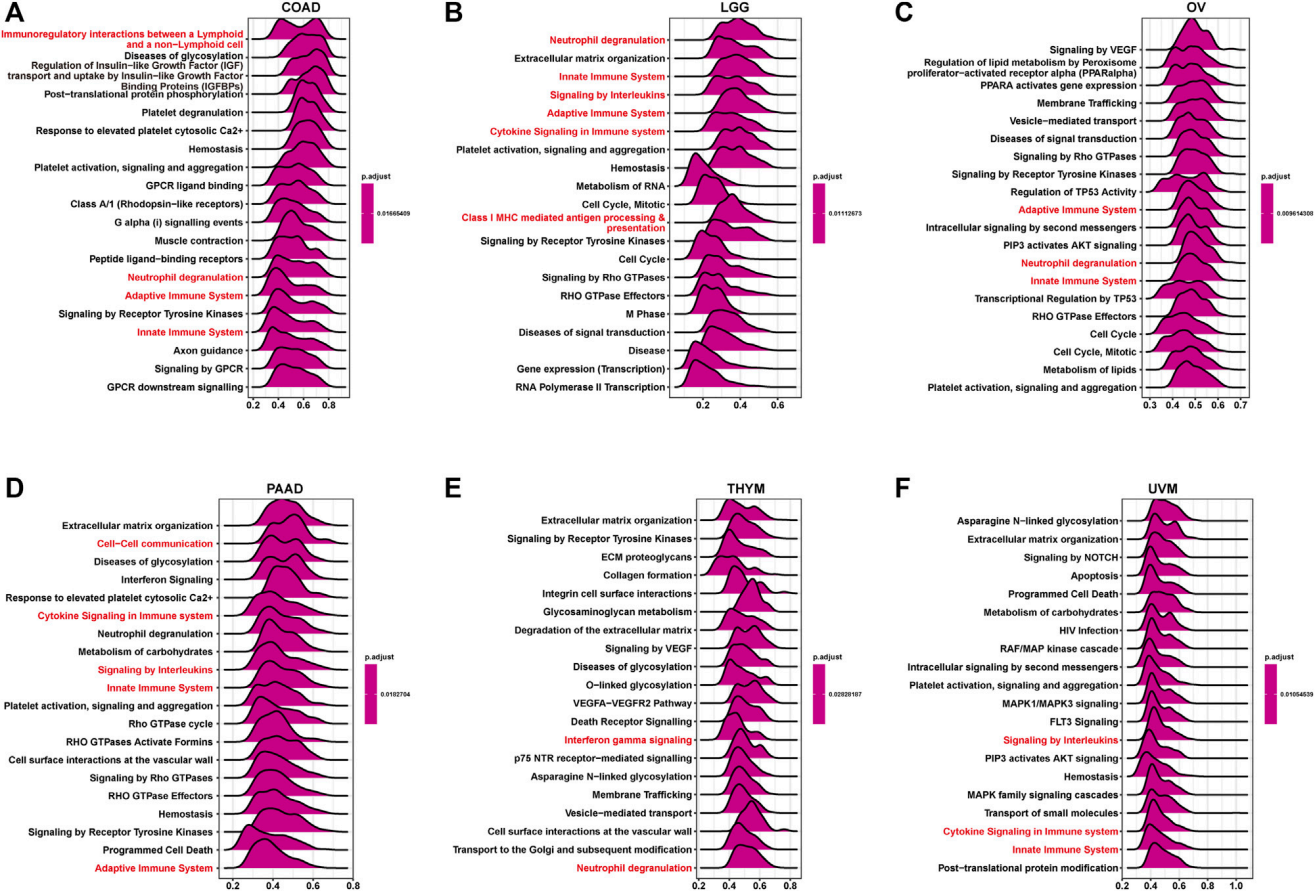
GSEA of TRPV4 in pan-cancer. **(A–F)** The top 20 GSEA results in indicated tumor types (NES ≥ 1.5, adjusted *p*-value < 0.05). Red indicates cell cycle-related or immune regulation-related terms.

GSEA based on the Reactome pathway database was used to predict pathways in which TRPV4 may be involved in pan-cancer. The GSEA results revealed that TRPV4 participates in immune regulation-related pathways in pan-cancer such as immunoregulatory interactions between lymphoid and non-lymphoid cells, the adaptive immune system, and the innate immune system (Figures 5A–F).

### TIME Analysis

**FIGURE 6:**
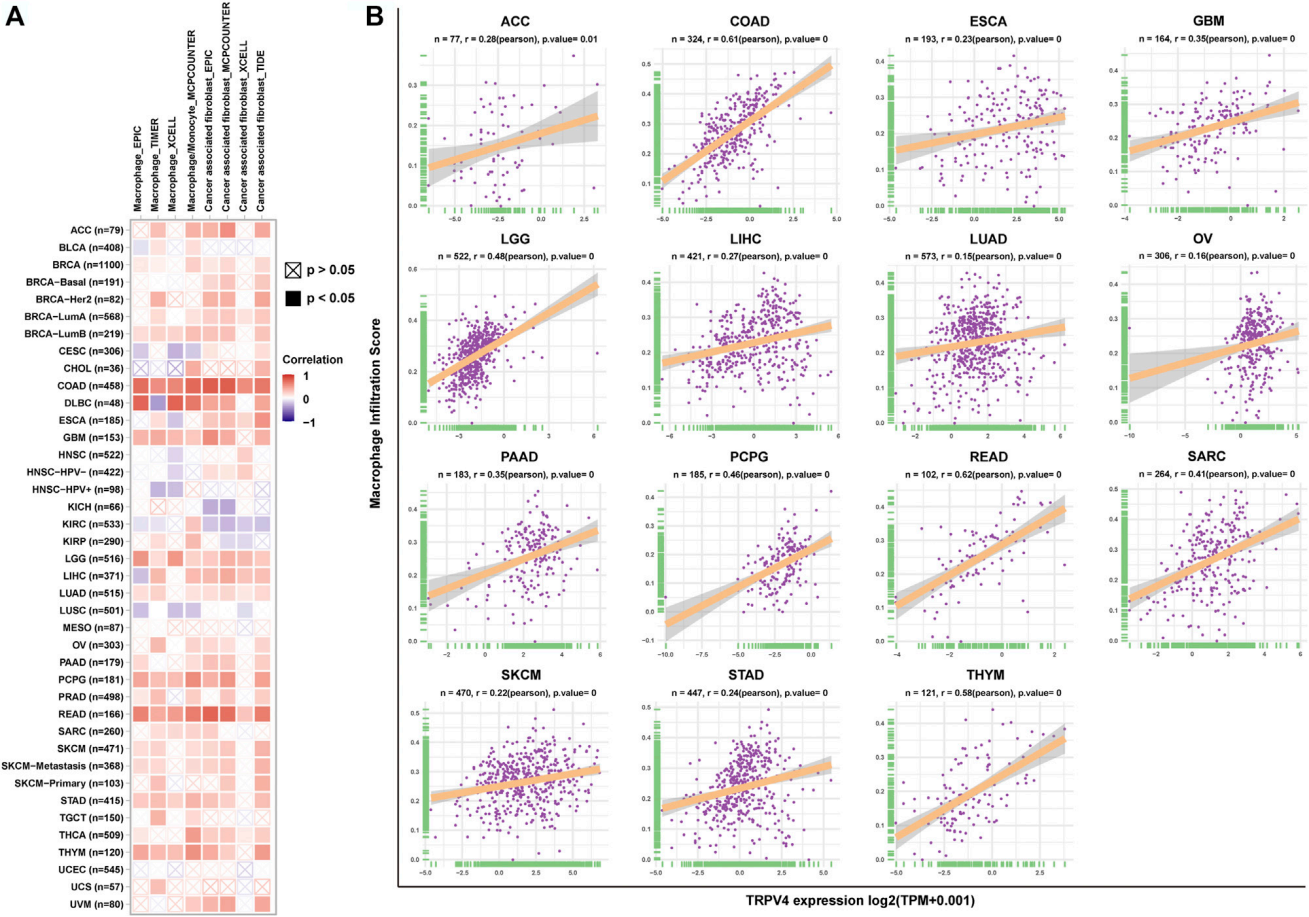
Immune cell infiltration analysis. **(A)** The correlation between TRPV4 expression and TAM and CAF infiltration levels in TCGA pan-cancer samples using the TIMER2 database. **(B)** The correlation between TRPV4 expression and TAM infiltration levels in TCGA pan-cancer samples using data from the ImmuCellAI database. The pearson’s correlation coefficient was calculated using R software.

Having predicted that TRPV4 is closely related to immune regulation pathways through GSEA analysis, we focused on the infiltration of immune cells in the tumor microenvironment. We found that TRPV4 expression was positively correlated with the infiltration level of TAMs and tumor associated fibroblasts (CAFs) in pan-cancer using the TIMER2 database (Figure 6A). To validate this result, we downloaded immune cell infiltration data from the ImmuCellAI database and performed a correlation analysis, obtaining the same result that TRPV4 expression was positively correlated with the level of TAMs in pan-cancer (Figure 6B).

**FIGURE 7:**
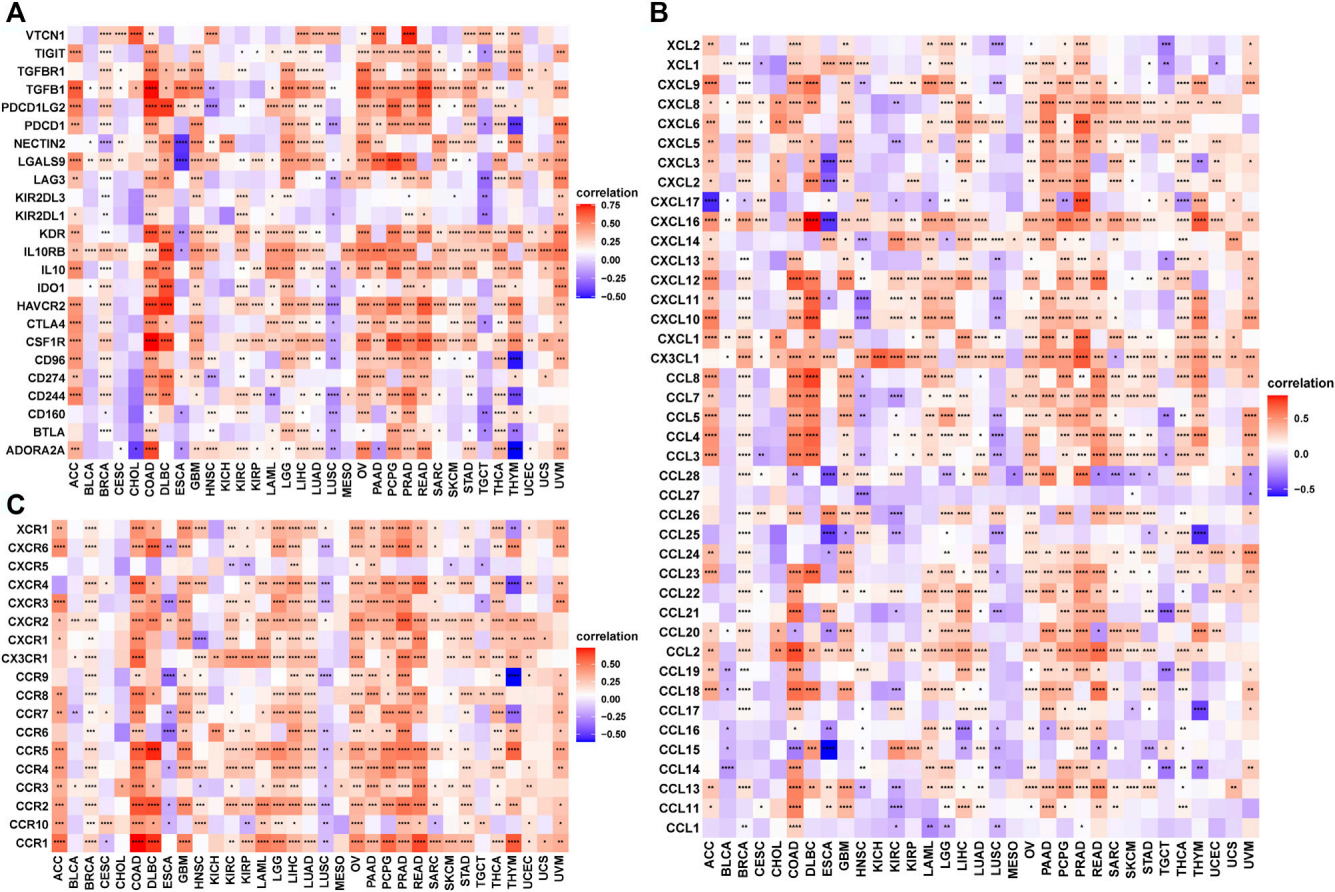
The correlation between TRPV4 and immunosuppressive genes. **(A)** Heatmap representing the correlation between TRPV4 expression and immunosuppressive status-related genes. **(B)** Heatmap representing the correlation between TRPV4 expression and chemokine genes. **(C)** Heatmap representing the correlation between TRPV4 expression and chemokine receptor genes. The pearson’s correlation coefficient was calculated using R software.

Next, we explored the correlation between TRPV4 with immune checkpoints, immunosuppressive genes, chemokines, and chemokine receptors. Immune checkpoint and immunosuppressive genes such as PD-L1, PD-1, CTLA4, LAG3, TIGIT, TGFB1, and TGFBR1 were positively correlated with TRPV4 in most tumors (Figure 7A). Chemokines and chemokine receptors such as CCL5, CCL5, CCR4, and CCR5 were also positively correlated with TRPV4 expression in most tumors (Figures 7B,C).

### Drug Resistance Analysis

**FIGURE 8:**
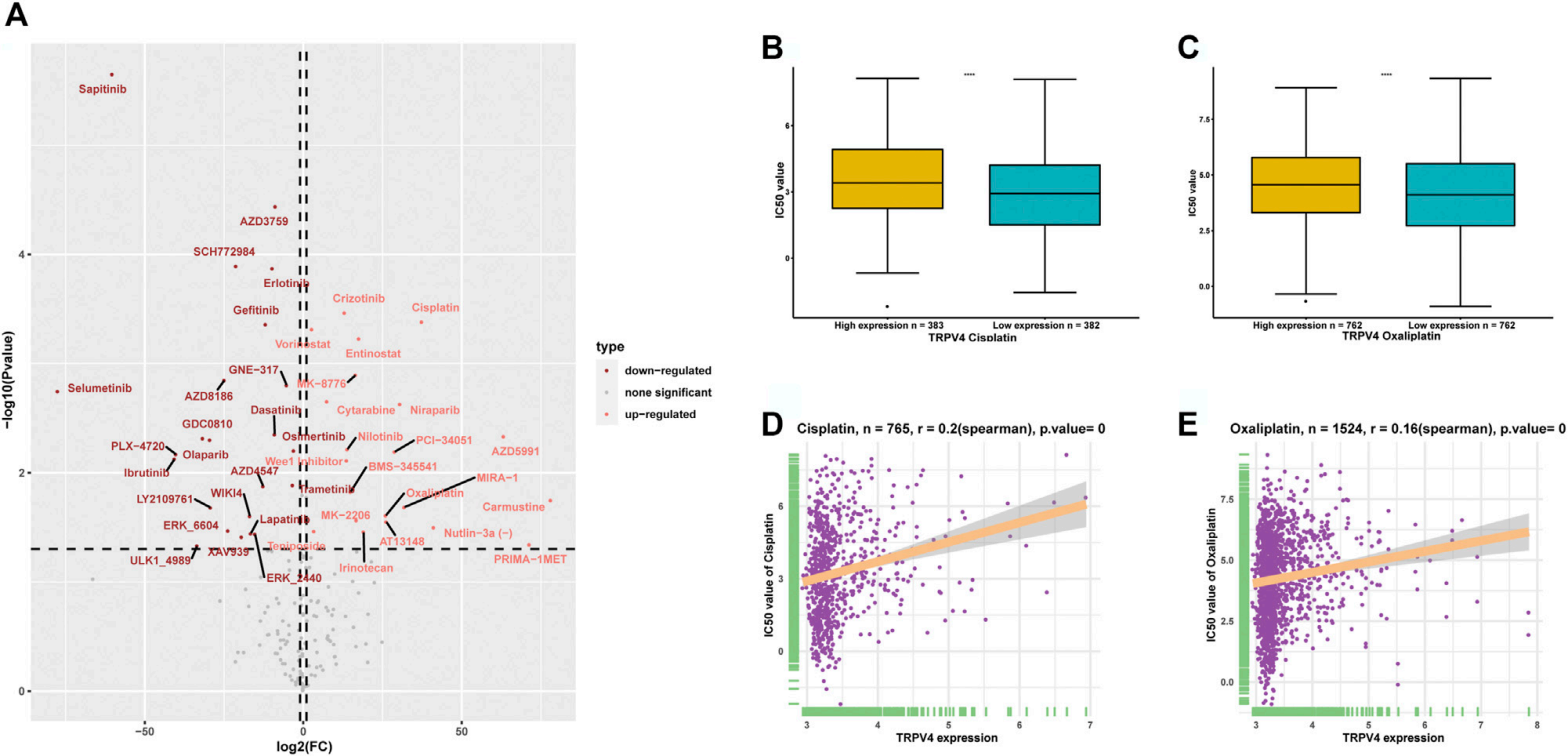
Anti-cancer drug sensitivity analysis. **(A)** Volcano map representing the differences in IC_50_ values of anti-cancer drugs between high- and low-TRPV4 groups. **(B)** IC_50_ values of cisplatin in high- and low-TRPV4 groups. **(C)** IC_50_ values of oxaliplatin in high- and low-TRPV4 groups. **(D)** The correlation between TRPV4 expression and IC_50_ values of cisplatin. **(E)** The correlation between TRPV4 expression and IC_50_ values of oxaliplatin. The spearman’s correlation coefficient was calculated using R software.

We downloaded the IC_50_ values of anti-cancer drugs and gene expression profiles in the relative cell lines from the GDSC database. To explore the influence of TRPV4 expression on the sensitivity of anti-cancer drugs, we divided the tumor cells into high- and low-TRPV4 groups and compared their IC_50_ values. We found that the IC_50_ values of several anti-cancer drugs decreased in the high-TRPV4 group, including sapitinib (an EGFR inhibitor) and selumetinib (a MEK1/2 inhibitor) (Figure 8A), indicating that patients exhibiting high TRPV4 expression levels are relatively sensitive to these anti-cancer drugs. In comparison, the IC_50_ values of platinum drugs such as cisplatin and oxaliplatin increased in the high-TRPV4 group (Figures 8A–C). Moreover, the IC_50_ values of cisplatin and oxaliplatin were positively correlated with TRPV4 expression (Figures 8D,E). These results indicate that patients exhibiting high TRPV4 expression levels may be resistant to cisplatin and oxaliplatin treatment.

## Discussion

TRPV4 is an omnipresent polymodally activated ion channel. Recent studies have shown that TRPV4 plays a role in a number of different functions in the body, including cancer. Moreover, the functions of TRPV4 are different or even opposite in different tumors. TRPV4 plays an oncogenic role in breast cancer, endometrial cancer, gastric cancer, oral squamous cell carcinoma, and COAD (Arbabian et al., 2020; Azimi et al., 2020; Fujii et al., 2020; Li et al., 2020; Wang et al., 2020) and a suppressor gene role in glioma (Huang et al., 2021). However—in many other tumor types—the function of TRPV4 remains unclear.

We first assessed TRPV4 expression using tumor tissue data from TCGA and normal tissue data from TCGA and the GTEx database. We found that TRPV4 was overexpressed in 19 cancer types, namely, BLCA, CESC, CHOL, COAD, DLBCL, ESCA, GBM, LAML, LGG, LUAD, LUSC, ovarian cancer, PAAD, READ, STAD, TGCT, THYM, UCEC, and UCS. In contrast, low TRPV4 expression was observed in only six cancer types, namely, ACC, KIRP, LIHC, PRAD, SKCM, and THCA. In COAD and ovarian cancer, TRPV4 was over-expressed; moreover, high expression levels of TRPV4 predicted worse OS in tumor patients. It has been reported that TRPV4 promotes the progression of COAD (Arbabian et al., 2020), while no reports on TRPV4 in ovarian cancer are available.

Tumor-acclimatized immune and stromal cells in the tumor microenvironment such as TAMs and CAFs play a vital role in accelerating tumor progression. The remodeling of immune cells by tumor cells can lead to immune escape (Komohara and Takeya, 2017). In our study, we predicted that TRPV4 is involved in immune regulation-related pathways using GSEA. Moreover, we proved that TRPV4 expression was positively correlated with TAMs and CAFs using two different methods: the TIMER2 and ImmuCellAI databases. Our results revealed that TRPV4 regulates the infiltration levels of TAMs and CAFs either directly or indirectly. Based on these results, we further explored the correlation between TRPV4 and immune checkpoints, immunosuppressive genes, chemokines, and chemokine receptors. Immune checkpoint and immunosuppressive genes such as PD-L1, PD-1, CTLA4, LAG3, TIGIT, TGFB1, and TGFBR1 were positively correlated with TRPV4 in most tumors. Chemokines and chemokine receptors such as CCL5, CCL5, CCR4, and CCR5 were also positively correlated with TRPV4 expression in most tumors. These results indicate that TRPV4 is closely associated with immune regulation. Patients with tumors exhibiting high TRPV4 expression levels may manifest an immunosuppressive status.

To explore the indicator function of TRPV4 in anti-cancer drug selection, we analyzed the association between TRPV4 expression and IC_50_ values of anti-cancer drugs using data from the GDSC database. We found that the IC_50_ values of several anti-cancer drugs, including sapitinib (an EGFR inhibitor) and selumetinib (a MEK1/2 inhibitor), decreased in the high-TRPV4 group, indicating that patients exhibiting high TRPV4 expression levels may be relatively sensitive to these anti-cancer drugs. In comparison, the IC_50_ values of platinum drugs—such as cisplatin and oxaliplatin—increased in the high-TRPV4 group. Moreover, the IC_50_ values of cisplatin and oxaliplatin were positively correlated with TRPV4 expression. These results indicate that patients exhibiting high TRPV4 expression levels may be resistant to cisplatin and oxaliplatin treatment.

## Conclusion

We conducted a comprehensive assessment of TRPV4, revealing its potential cancer-promoting effect as well as its role as an indicator of patient prognosis. Importantly, high TRPV4 expression often indicates tumor immunosuppression, which may render immune checkpoint inhibitors unsuitable for treatment. Finally, we found that patients exhibiting high TRPV4 expression levels may be resistant to cisplatin and oxaliplatin treatment and sensitive to sapitinib (an EGFR inhibitor) and selumetinib (a MEK1/2 inhibitor) treatment. The impact of TRPV4 on the progression of numerous cancers requires a comprehensive understanding of its role as a biomarker for patient prognosis based on its broad influence on anti-cancer drug efficacy.

## Data Availability

The datasets presented in this study can be found in online repositories. The names of the repository/repositories and accession number(s) can be found in the article/Supplementary Material.

## Abbreviations

BLCA, bladder urothelial carcinoma; BRCA, breast invasive carcinoma; CNA, copy number alteration; COAD, colon adenocarcinoma; DFI, disease-free interval; DLBCL, diffuse large B-cell lymphoma; DSS, disease-specific survival; ESCA, esophageal carcinoma; GDSC, genomics of drug sensitivity in cancer; KICH, kidney chromophobe; KIRC, kidney renal clear cell carcinoma; LGG, low-grade glioma; LIHC, liver hepatocellular carcinoma; LUAD, lung adenocarcinoma; LUSC, lung squamous cell carcinoma; OS, overall survival; PFI, progression-free interval; PRAD, prostate adenocarcinoma; READ, rectum adenocarcinoma; SKCM, skin cutaneous melanoma; STAD, stomach adenocarcinoma; TAM, tumor-associated macrophage; TCGA, the Cancer genome atlas; TGCT, testicular germ cell tumors; THCA, thyroid carcinoma; UCEC, uterine corpus endometrial carcinoma; UCS, uterine carcinosarcoma.
