# Supplementary figures and images for: TRPV4 is a Prognostic Biomarker that Correlates with the Immunosuppressive Microenvironment and Chemoresistance of Anti-Cancer Drugs
Source: Front Mol Biosci. 2021 Jun 28;8:690500. doi: 10.3389/fmolb.2021.690500 (PMC8273915; doi:10.3389/fmolb.2021.690500)

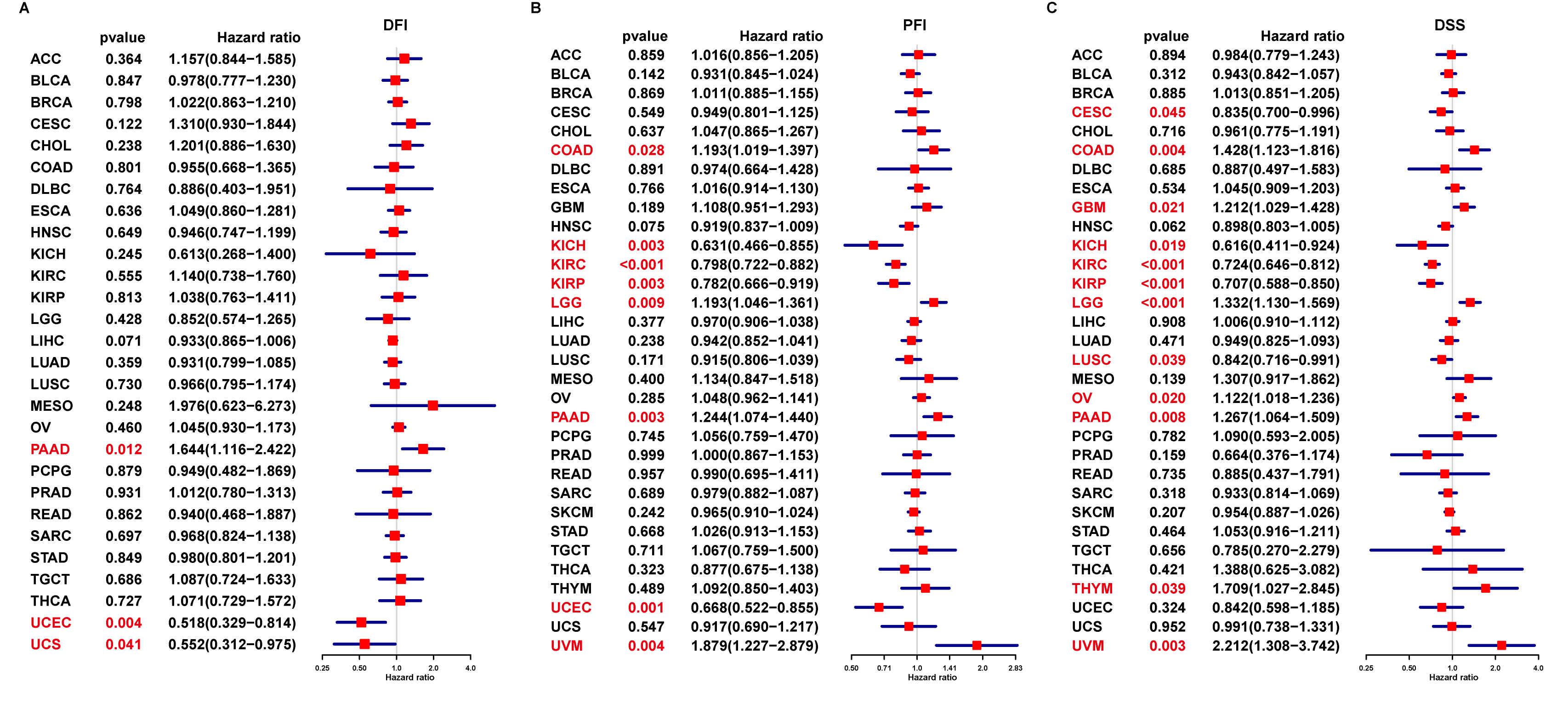

Supplement: Supplementary file 1 [file Image1.tif]
